# Protocol: an improved and universal procedure for whole-mount immunolocalization in plants

**DOI:** 10.1186/s13007-015-0094-2

**Published:** 2015-10-28

**Authors:** Taras Pasternak, Olaf Tietz, Katja Rapp, Maura Begheldo, Roland Nitschke, Benedetto Ruperti, Klaus Palme

**Affiliations:** Faculty of Biology, Institute of Biology II/Molecular Plant Physiology, University of Freiburg, Freiburg, Germany; BIOSS Centre for Biological Signaling Studies, University of Freiburg, Freiburg, Germany; Freiburg Institute for Advanced Studies (FRIAS), University of Freiburg, Freiburg, Germany; Department of Agronomy, Food, Natural Resources, Animals and Environment, DAFNAE, University of Padova, Agripolis, Viale dell’Università, 35020 Legnaro, Padova Italy; Center for Biological Systems Analysis, University of Freiburg, Freiburg, Germany

**Keywords:** Immunolocalization, Tissue multi-protein expression, Whole-mount, 3D reconstruction, Protein–protein interaction

## Abstract

Rapid advances in microscopy have boosted research on cell biology. However sample preparation enabling excellent reproducible tissue preservation and cell labeling for in depth microscopic analysis of inner cell layers, tissues and organs still represents a major challenge for immunolocalization studies. Here we describe a protocol for whole-mount immunolocalization of proteins which is applicable to a wide range of plant species. The protocol is improved and robust for optimal sample fixation, tissue clearing and multi-protein staining procedures and can be used in combination with simultaneous detection of specific sequences of nucleic acids. In addition, cell wall and nucleus labelling can be implemented in the protocol, thereby allowing a detailed analysis of morphology and gene expression patterns with single-cell resolution. Besides enabling accurate, high resolution and reproducible protein detection in expression and localization studies, the procedure takes a single working day to complete without the need for robotic equipment.

**Electronic supplementary material:**

The online version of this article (doi:10.1186/s13007-015-0094-2) contains supplementary material, which is available to authorized users.

## Background

Multicolor immunolocalization and imaging approaches are increasingly used in plant biology for a variety of different purposes including analysis of protein localization and protein–protein interactions in specific tissue contexts [1], tracking of cell anatomy [2], visualizing tissue and cellular distribution of specific low molecular weight molecules (i.e. hormones such as auxin) [3] and recording signaling events at the organelle subcellular level. In plants robust and reliable techniques are highly required for the accurate whole-mount visualization of subcellular protein localization in relatively thick specimens, in a well preserved tissue structure to analyze patterns of gene expression in developmental studies. Current techniques for the whole-mount visualization of protein expression and subsequent three-dimensional (3D) imaging include fluorescent protein localization [4] and immunolocalization with antibodies on Arabidopsis seedlings [5, 6]. These methods work relatively well on roots of very young Arabidopsis seedlings, where tissue penetration is facilitated but they are currently limited with respect to the depth of penetration within the tissue(s) and to the resolution that can be achieved. Confocal laser scanning microscopy of plant tissues allows analysis of relatively thin and semitransparent organs, while penetration and optical sectioning for 3D reconstruction of relatively thick specimens is limited so that cellular and intracellular details are usually difficult to resolve also when two-photon confocal microscopes are used. Particularly, the simultaneous localization of nucleic acids (DNA, RNA) and of fluorescently labeled proteins (through translational fusions) are difficult to perform in depth on tissues, even if they have been cleared to reduce background fluorescence. Similarly, the use of antibodies labeled with fluorescent dyes for immunolocalization studies suffer from poor tissue penetration or bad tissue preservation after harsh chemical treatments which are necessarily performed to improve penetration of antibodies into deeper cell layers. In addition, currently available whole-mount protocols [5, 6] consist of a large number of steps and are sometimes poorly reproducible due to limitations with respect to antibody penetrance and tissue preservation [7, 8]. We have systematically analyzed critical parameters for tissue fixation, improved cell permeation techniques and developed a protocol for reproducible visualization of internal tissue structures of different plant organs (e.g. siliques, ovules, roots) at all stages of development without requirement for sectioning.

Tissue fixation has been found as the most crucial step: effective and rapid penetration of the fixative in the inner cell layers has a primary importance for all further steps. Therefore an effective combination of vacuum with a detergent is crucial for successful fixation. The plant cuticle is an extracellular hydrophobic layer that covers the aerial epidermis of all land plants, providing protection against desiccation. In our protocol hot methanol (up to 60 °C) has been implemented as an effective way for permeabilization of the cuticle and increasing tissue permeabilization, especially in dense organs in the inner cell layers.

We show that the protocol is fast, simple, suitable for automation, and presents a highly valuable, robust tool for the study of the cellular organization of a wide range of plant tissues. In addition the improved method allows simultaneous staining of nucleic acids and of proteins, and enables obtaining high resolution images of a quality suitable for 3D confocal reconstruction of cellular gene expression networks within a plant organ. We demonstrate the usefulness of this protocol for the characterization of auxin transport routes in a number of dicotyledonous and monocotyledonous plant species, during ovule reproductive organ development, and cytoskeleton labeling during mitosis. The reported protocol allows robust immunolabeling of different tissues in a wide range of plant species at high penetration depth, independently from tissue transparency and density, enabling better resolution and 3-D reconstruction for digital atlas of whole plants organs (roots, leaf etc.) [9].

## Methods

### Reagents and solutions

Antifade mounting medium: Fluoromount G (refractive index 1.393; Southern Biotech, cat. no. 0100^-^01) or ProlongGold (refractive index 1.47; http://products.invitrogen.com/ivgn/product/P36930);

Blocking solution: 2 % albumin fraction V BSA (Carl Roth, cat. no. 8076.2) in 1 × MTSB;

Calcofluor white (BR 28, Sigma, cat. no. F3543) (0.4 mg/l in 10 mM Tris-HCl pH 9.2) (dilute from 1 mg ml^−1^ stock in DMSO);

Cell wall digestion enzymes: 0.2 % Driselase (Sigma, cat. no. D9515), 0.15 % Macerozyme (Duchefa, cat. N M8002.0010) in 2 mM MES (Sigma, cat. no M3671), pH 5.0;

Nuclear stain: DAPI (4′,6-diamidino-2-phenylindole dihydrochloride; Sigma, cat. no. D9564) (0.2 mg/l) in water (dilute from 1 mg ml^−1^ stock in water). Note: Dissolve DAPI in water at a concentration of 1 mg/ml and dilute it before use to 2 μl in 10 ml. A 1 mg/ml solution is stable for at least 1 year at 4 °C;

Fixative solution: 2 % paraformaldehyde (Merck, cat. no. 1040051000) in 1× MTSB supplemented with 0.1 % Triton X-100 (Carl Roth, cat. no. 3051.2); Solution preparation: 2 g of Para-formaldehyde is dissolved in 20 ml of water (10 % stock solution) by stripping and slightly warming to 65-70 °C and addition of one drop of 1 M KOH. The stock solution can be stored in 2 ml aliquots at −20 °C. Prior to usage it is diluted to 2 % paraformaldehyde in using 2× MTSB and water to reach 1× MTSB (final concentrations);

Methanol (Carl Roth, cat. no. 4627.2) for tissue fixation, clearing and cuticle solubilization;

MTSB (microtubule-stabilizing buffer): Preparation of stock solution (2× MTSB): 15 g PIPES (FW 302.4; Roth, cat. no. 9156.3), 1.90 g EGTA (FW 380.4; Roth, cat. no. 3054.2), 1.22 g MgSO_4_·7H_2_O (FW 246.48; Carl Roth, cat. no. 8283.1) and 2.5 g KOH (FW 56.11; Carl Roth, cat. no. 6751.1) are dissolved in a total of 500 ml water at pH 7.0 (adjusted with 10 M KOH);

Permeabilization buffer: 3 % non-ionic detergent IGEPAL CA-630 (Sigma, cat. no. I3021) (Similar to Nonidet P-40, which is no longer commercially available) plus 10 % dimethylsulfoxide (DMSO) (Carl Roth, cat. no. 4720.2) in 1× MTSB buffer;

Primary antibody solution: the primary antibody solution is prepared in blocking solution; the optimal antibody concentration must be determined experimentally and can vary between 1:20 and 1:1000;

Propidium Iodine (PI, Sigma, cat. no. P4170) (1 mg/l) in 10 mM Tris–HCl, pH 7.5 diluted from 4 mg/ml stock in water;

RNAse solution (0.1 mg/ml) in 10 mM Tris–HCl, pH 7.5 (Sigma, cat. no. R5000) prepared from 1 mg/ml stock, diluted in water;

Secondary antibody solution (preparation in 1× blocking buffer with 1:500 dilution immediately before use).

### Equipment

Shaker for gentle shaking during fixation.

Agilent slides (G2534-60530 or G2534-60535, with 8 or 4 rubber frames (Additional file 1) for whole plant/organ labeling;

Confocal microscope (recommended; alternatively, epifluorescent microscope);

Conical tubes (Greiner) 15 and 50 ml;

Cover glasses: 0.17 mm thick; 24 × 40 mm (CarlRoth, cat N. 1870.2); we recommend for high resolution microscopy always cover-glasses of defined thickness 0.17 mm ± 0.01 or 0.005 mm.

Incubator (37 °C);

Forceps (Carl Roth, cat. no. K341.1);

Humid chambers are prepared from 90 mm Petri dishes with wet absorbent paper inside;

Micropipettes;

Microscope slides for mounting of specimens after labeling;

Parafilm strips;

Poly-l-Lysine Coated Microscope Slides (e.g. from Polysciences, cat. no. 22247-1) or home-made slides coated with 10 % Poly-l-Lysine solution were used when immunolocalization experiments were performed on protoplasts or suspension cells;

Scalpel (Carl Roth, cat. no. 3607.1 or 3596.1);

Stereo microscope;

Vacuum pump (water-jet type or comparable) with a desiccator;

Well Suspension Culture Plate 12 or 24 well (Greiner, cat. no. 665102 or 662102).

### Protocol: procedure steps

**Fig. 1:**
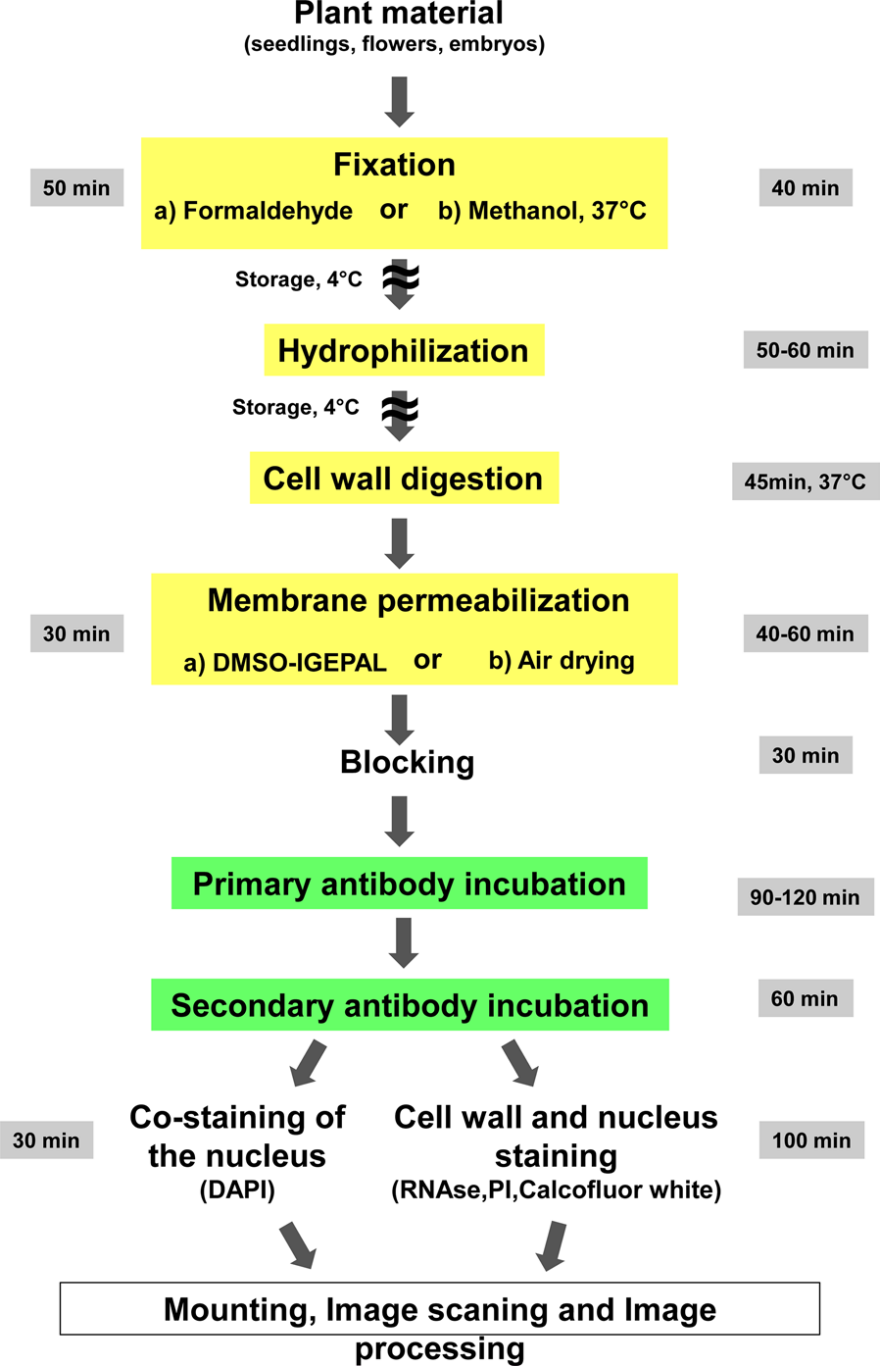
Work flow diagram. The different steps of the immunolocalization protocol are indicated in *boxes* linked by *arrows* and colored in *yellow* for fixation and permeabilization steps. Antibodies incubation steps are *boxed* in *green*. The time required for each step is indicated on the side of the *box*. Possible pause points are indicated in the diagram by *wave line across the arrows*

An overview of the main steps of the procedure is presented in Fig. 1, with the indication of the time required to perform each step and of steps where the procedure can be stopped. The whole procedure is described step-by-step below by giving a detailed description followed by background notes with comments. The recommend volumes have been calculated for 24 wells plates and 8 rubber frames slides.

#### Step 1: Fixation

##### Fixation A (Formaldehyde)

Timing: 50–60 min.

- Place explants in at least 1.5 ml of 2 % formaldehyde in 1× MTSB buffer supplemented with 0.1 % Triton, pH ~ 7, (ratio fixative/explants 10:1).
- Apply vacuum infiltration for 2–3 min and then (slowly) release vacuum. Repeat it once again. The fixation starts only after fixative penetrates (or air will be back to the desiccator).
- Check if explants have sunk at the bottom, and continue fixation for 40 min under gentle shaking at 37 °C.
- Wash samples in 2 ml of distilled water ~10 min.

#### Alternative procedure

##### Fixation B (methanol)

Timing: 40 min.

- Place explants in 1.5 ml of 100 % p.a. methanol for 20 min and incubate at 37 °C.
- replace with 0.8 ml of fresh 100 % p.a. methanol (60 °C), incubate vials for 3 min and gradually add water till final concentration of methanol reaches 20 % (ca 3.2 ml water). Thereafter transfer explants/plants to a new vial with water. In our hands methanol preserved protein structure and has allowed combining successfully tissue clearing with cuticle solubilization.

*Comments* The goal of fixation is to maintain the cellular structure as intact as possible. Tissue fixation can be performed by two different ways (reported above as fixation A and B, respectively) depending on the proteins of interest. Fixation with formaldehyde (fixation A) crosslinks proteins with cellular components which preserve tissue and cell morphology. Rapid penetration of the fixative into the cells is crucial for proper fixation. This is assured by vacuum infiltration of the fixative, containing 0.1 % Triton (surfactant), into the tissue. Freshly prepared 2 % formaldehyde solution from para-formaldehyde powder is used for this purpose, giving best results. If commercially available 37 % formaldehyde stock solution is used, the instability of formaldehyde in solution and its polymerization during long term storage may hamper results and has to be taken into account. Specimens should be fixed in a multiwell culture plate with a large surface to enable efficient gas removal through vacuum application during fixation procedure. In many cases methanol fixation (fixation B) alone is enough to preserve protein and cellular structure and has allowed in our hands combining successfully tissue clearing with cuticle solubilization, thus providing a good, faster and easier alternative to formaldehyde fixation. Absorbed methanol is oxidized within the plant cell to formaldehyde and formic acid [10].

In general, from our experience, methanol works well for membrane proteins. In addition, aerial parts of plants (leaves of certain species) have a highly hydrophobic cuticle to prevent water loss. In order to allow antibodies to penetrate inside cells, the cuticle needs to be solubilized. This can be achieved by treatment with methanol which solubilizes the majority of the cuticle and other waxes. We also experienced that methanol treatment also improved antibody penetration in the mature part of the root. Finally, chlorophyll, as a potential source of auto-fluorescence, is readily removed by methanol treatment as well. However, one should also consider that some epitopes are very sensitive to methanol and may be not accessible anymore for antibody binding so a comparison of the two fixative methods should be considered.

#### Step 2: Cuticle solubilization and tissue clearing—hydrophilisation

Timing: 50–60 min.

- Replace water (from fixation A) with ~0.8 ml of 100 % p.a. methanol (60 °C) and incubate for ~5–10 min or, from fixation B, directly proceed to the following step.
- Gradually decrease alcohol concentration by adding every 2 min 100–200 µl of water until the final alcohol concentration reaches ~20 % (this corresponds to the addition of 3.2 ml of water).
- Wash twice for 5 min each in water.
- Transfer plants to the agilent slides pre-loaded with 60 µl of water.

*Comments* Gradual addition of water is important for preserving the structure of tissues/organs.

#### Step 3: Digestion of cell walls

Timing: 45 min.

- Add 60 µl of the cell wall digestion solution into each well/frame (0.2 % Driselase and 0.15 % Macerozyme in 2 mM MES, pH 5.0).
- Incubate for 30–40 min. at 37 °C.
- Wash 1 × 4 min with 100 µl of the 1× MTSB pH 7.0.

*Comments* In contrast to animal cells, plant cells are surrounded by a rigid cell wall, which needs to be at least partially digested for efficient antibody penetration. Therefore tissues are incubated with cell wall degrading enzymes. In addition, dense tissues specifically need to be macerated for effective antibody penetration into deeper layers. In the majority of published protocols Driselase is used dissolved in 1× MTSB buffer with pH of approximately 7.0 [3]. These conditions are suboptimal, because Driselase has quite low cell maceration activities and its pectolytic and cellulolytic activities have an optimum pH ranging from 4.0 to 6.0 and from 3.0 to 5.0, respectively [11]. In order to improve the cell wall digestion and increase tissue maceration a mixture of Driselase and Macerozyme R10 was used in MES buffer with pH 5.0. This treatment is gentler and results reproducibly in excellent preserved tissues.

#### Step 4: Membrane permeabilisation

Timing: 30 min.

- Add 60 µl of the membrane permeabilisation solution (3 % IGEPAL C630, 10 % DMSO in 1× MTSB) and incubate for 15–20 min at 37 °C.
- Wash 4 times with 1x MTSB for 3 min each.

*Comments* After partial digestion of cell walls, the cellular membranes must be permeabilized. Membrane permeabilization creates pores in membranes, which allow the antibody to penetrate. For this purpose treatment with a mixture of DMSO and the detergent IGEPAL CA-630 was applied. This treatment allows efficient and reproducible antibody penetration. As an alternative to treatment with IGEPAL/DMSO, one can completely dry the tissue on the slide. This option is favorable for cell monolayer cultures (see supplementary protocol for suspension cells), but also can help tissue permeabilization in the case of whole organs.

#### Step 5: Blocking

Timing: 30 min.

- Add 60 μl of blocking buffer to each frame and incubate for 20 min.

*Comments* the goal of the blocking step is to minimize non-specific antibody binding. The minimal duration of the blocking is 20 min., however, in some cases (when background noise is high), it can be extended to up to 2 h.

#### Step 6: Primary antibody incubation

Timing: 90–120 min.

- Replace blocking solution with 60 µl of the primary antibody solution and incubate for 1–2 h at 37 °C;
- Wash 2 × 5 min with 100 µl of the 1× MTSB.

*Comments* Do not mix solution during incubation with the primary antibody.

Antibodies used for immunostaining should be always affinity purified. According to our experience it is not advisable to use crude sera due to cross-reactivity with multiple proteins. Best results are obtained with antibodies against epitope tags (HA, Myc) or GFP, but this is only suitable for genetically modifiable species like Arabidopsis. It is absolutely necessary to test antibody specificity in Western blots. A loss of function mutant where the protein of interest is absent, if available, should be ideally used as a negative control. As a negative control, samples should be also incubated in the presence of pre-immune serum.

#### Step 7: Secondary antibody incubation

Timing: 60 min.

- Add 60 µl of the secondary antibody solution, and incubate for 1–2 h at 37 °C;
- Wash 3 × 5 min with 1x MTSB.

*Comments* Do not mix solution during incubation with the secondary antibody. The choice of fluorophore with which secondary antibodies are conjugated depends primarily on the task of investigation. Fluorophores are differing in terms of brightness, photobleaching and chemical stability. Many of the most popular secondary antibodies are Alexa conjugated (InVitrogen). However, recently new DyLight antibodies have been developed (InVitrogen, Agrisera, Abcam). DyLight ^®^ conjugated secondary antibodies exhibit higher fluorescence intensity, photo stability and water solubility and remain fluorescent from pH 4 to pH 9. Additionally, the water solubility of the DyLight^®^ fluorophores allows a high dye-to-antibody ratio to be achieved without causing precipitation of conjugates.

For protein co-localization studies up to four primary and secondary antibodies can be used simultaneously, but they should be raised in different animals to avoid cross-reactivity.

#### Step 8: Co-staining of the nucleus

Timing: 15 min.

- Add 100 µl of the DAPI containing solution (0.2 mg/l) and incubate for 10 min;
- Wash 3 × 5 min with 100 ml of distilled water.

#### Step 8 (alternative): Cell wall and nucleus staining

Timing: 50 min.

- Incubate in 10 mM Tris–HCl, pH 7.5 for 10 min;
- Incubate in 100 µl of the RNAse solution in 10 mM Tris–HCl, pH 7.5 for 30 min at 37 °C;
- Wash 1 × 5 min with 100 µl of 10 mM Tris–HCl, pH 7.5;
- Incubate in 100 µl of the propidium iodine solution (0.4 mg/l) in 10 mM Tris–HCl, pH 7.5 for 10 min at 37 °C;
- Wash with water for 10 min;
- Incubate in 100 µl of the 10 mM Tris–HCl, pH 9.2 for 10 min;
- Incubate in 100 µl of the calcofluor white solution in 10 mM Tris, pH 9.2 for 20 min;
- Wash 2 × 5 min in the 10 mM Tris–HCl, pH 9.2.

*Comments* In order to show the proteins of interest in a cellular and organ continuity, additional staining of cell walls and of nuclei with calcofluor white and propidium iodine, respectively, might be wishful. This procedure does not affect the detection of proteins. Calcofluor white requires an alkaline pH for binding to the cell wall. We recommend keeping pH at 8.5–9 also in the mounting solution by mixing 70 % of antifade medium with 30 % of 500 mM Tris–HCl, pH 9.2 (350 µl antifade medium + 150 µl 500 mM Tris–HCl, pH 9.2).

#### Step 9: Mounting

Transfer seedlings to microscopic slides with a jacket containing antifade medium, cover samples with a cover slip and store them in the fridge/cold-room (approximately 5 °C).

*Comments* To prepare samples for microscopy, they are embedded in commercially available antifade solutions like Fluoromount G (Southern Biotech) or Prolong^®^ Gold reagent (Invitrogen). These solutions satisfactorily protect samples from photo-bleaching. We highly recommend to match as near as possible the refractive index of the mounting medium to the refractive index of the immersion medium used for the microscopical imaging to avoid optical artifacts, strong fluorescence emission and signal loss due to a mismatch. One also can use home-made antifade solutions, containing glycerol (50 %), *N*-propyl gallate (15 mg/ml) (final concentration) and H_2_O (50 %). For long term storage of samples addition of 0.1 % sodium azide to the anti-bleaching solution is mandatory. In order not to damage the samples we suggest to mount specimens after immunolocalization on microscopic slides with pre-inserted 120 µm spacer made from TVC isolation tape. The tape is cut in small stripes and pasted on the slide before samples insertion. Appropriate thickness of the jacket avoids tissue pressing and enables to reconstruct 3D images from the organs/seedlings. For Arabidopsis whole mounted seedlings a 100 μm thick spacer is sufficient to keep the original 3D structure.

### Comments and concluding remarks

The reported protocol for immunolocalization allows researchers to study metabolites, nucleic acids and protein localization in virtually any plant species and organs in relatively thick specimens speeding up throughput and resolution of protein localization studies also in non-model plants. The presented methodologies significantly improve the accuracy and resolution of protein detection in expression and localization studies and do not have a limit regarding tissue type. Manual sectioning can be avoided and 3D reconstruction can be easily done. Its shortest version takes only 7 h to complete without the need for robotic equipment, as shown in Fig. 1. Additional applications of the protocol are also provided for immunolocalisation on isolated plant cells and protoplasts and for 3D reconstuction (Additional file 2).

**Fig. 2:**
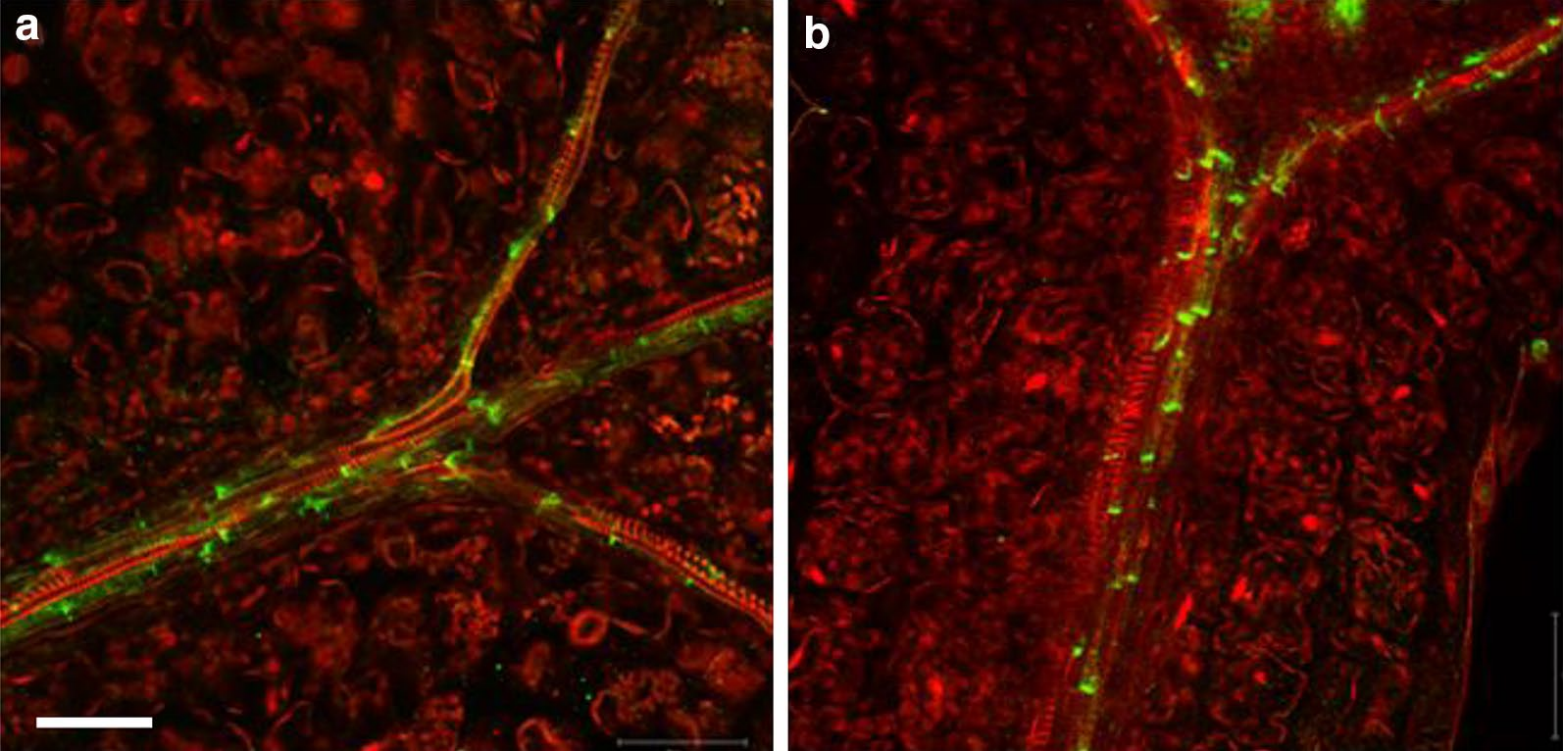
PIN1 protein localization in cotyledons and hypocotyls of Arabidopsis seedlings. Four days old seedlings were fixed for 20 min in methanol and subjected to the standard immunolocalization procedure as described. Anti-PIN1 mouse monoclonal primary antibody (clone 10A7), diluted 1: 50. ALEXA Fluor ^®^ 488 conjugated goat anti-mouse IgG (Invitrogen) was used as secondary antibody diluted 1:800. Co-staining with DAPI visualized nuclei (*red*). **a** Cotyledon; **b** hypocotyl; *Scale bar* 20 µm

**Fig. 3:**
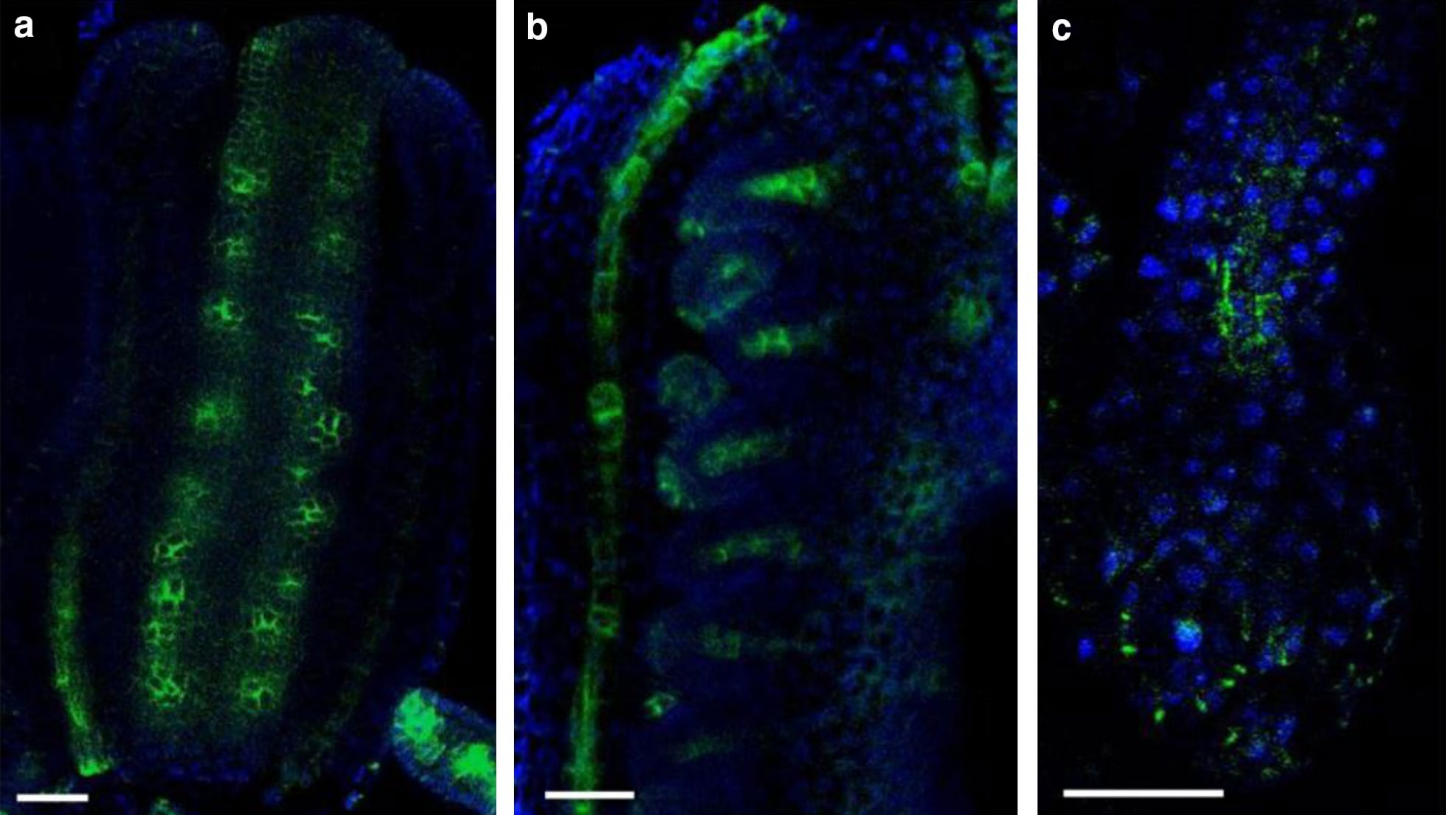
Auxin efflux carrier PIN1 localization in Arabidopsis flower organs. Whole siliques were fixed in formaldehyde and treated for 20 min with methanol. Anti-PIN1 mouse monoclonal primary antibody (clone 10A7), diluted 1:50 and ALEXA Fluor ^®^ 488 goat anti-mouse IgG as secondary antibody (Invitrogen) diluted 1:800 were used. Co-staining with DAPI visualizes nuclei (*blue*). **a** Arabidopsis silique, stage 1; **b** Arabidopsis silique, stage 2; **c** Isolated ovules. *Scale bar* 20 µm

**Fig. 4:**
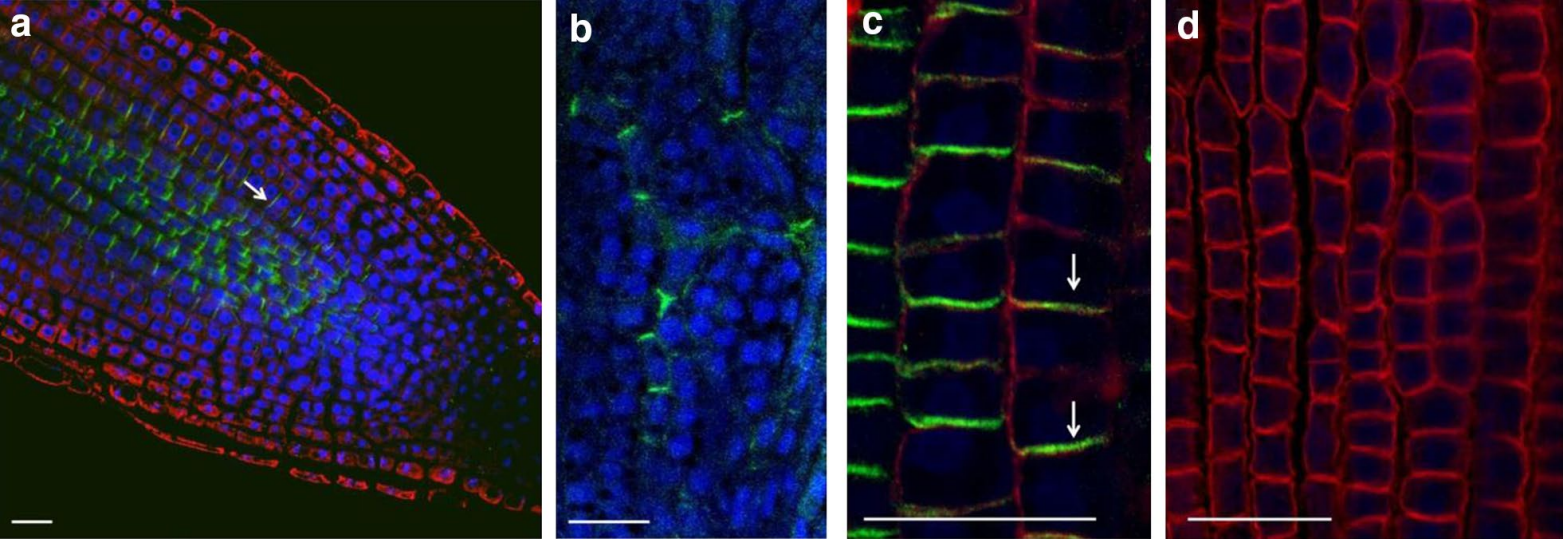
Protein immunolocalization in *Medicago sativa* L. and *Lycopersicum esculentum* L. Plants were fixed for 30 min in formaldehyde. Anti-PIN1 mouse monoclonal primary antibody (clone 10A7) diluted 1:50 plus Alexa Fluor^®^488 goat anti-mouse IgG as secondary antibody diluted 1:800 (shown in *green* *color*) and H^+^-ATPase (AS07 260) rabbit primary antibody plus Alexa Fluor^®^ 555 goat anti-rabbit IgG as secondary antibody diluted 1:800 (shown in *red color*) were used. Nuclei are visualized by co-staining with DAPI (*blue*). *Scale bar* 20 µm. *White arrows* show polar PIN1 localisation. **a** *Medicago sativa* roots; **b** *Medicago sativa* leaf;** c**,** d**- Lycopersicum esculentum root

**Fig. 5:**
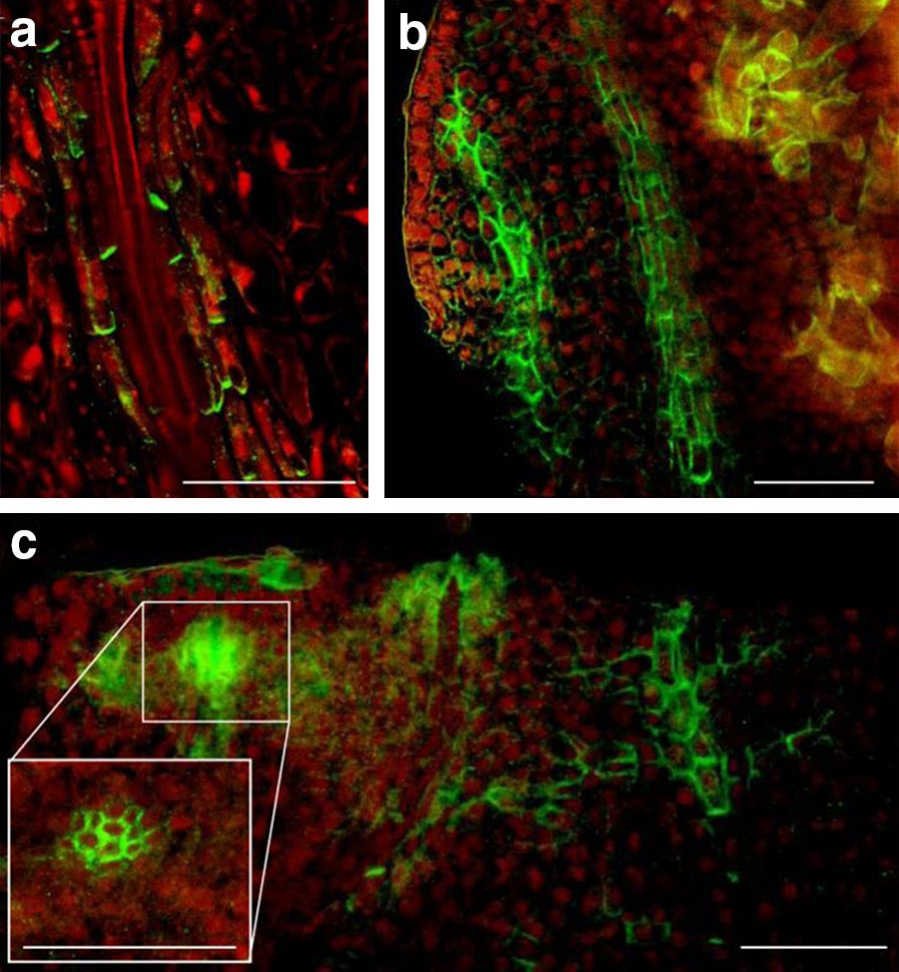
PIN1 protein localization in *Hedera helix* stem, leaf and flowers. Explants were fixed in formaldehyde for 30 min. Anti-PIN1 mouse primary antibody (clone 10A7) diluted 1:50 plus Alexa Fluor^®^ 488 goat anti-mouse as secondary antibody diluted 1:800 was used. Co-staining with DAPI visualizes nuclei (shown as artificial color in *red*). *Scale bar* 20 µm. **a** stem; **b** leaf; **c** flowers initial

**Fig. 6:**
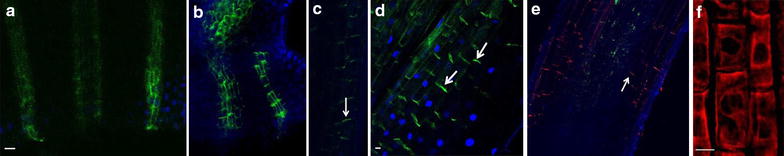
Protein immunolocalization in different *Triticum aestivum* organs. Three days old wheat seedlings were fixed for 30 min in formaldehyde. Anti-PIN1 mouse monoclonal primary antibody (clone 10A7) diluted 1:50 and Alexa Fluor^®^ 488 goat anti-mouse IgG as secondary antibody diluted 1:800 were used (shown in *green color*) (**a**–**e**); anti-PIN2 Guinea pig primary antibody plus Goat anti-Guinea pig IgG Alexa Fluor^®^ 647 conjugate as secondary antibody diluted 1:800 (shown in *red color*) (**e**) and anti-BIP2 (AS09 615) rabbit primary antibody plus Goat anti-rabbit IgG DyLight^®^ 549 conjugate (AS09 642) as secondary antibody diluted 1:3000 (shown in *red color*) (**f**) were used. Co-staining with DAPI visualizes nuclei (*blue*). **a** leaf; **b** meristem; **c** coleoptile; **d**–**f** roots. *Arrows* point polarly located PIN1 and PIN2 proteins. *Scale bar* 20 µm

**Fig. 7:**
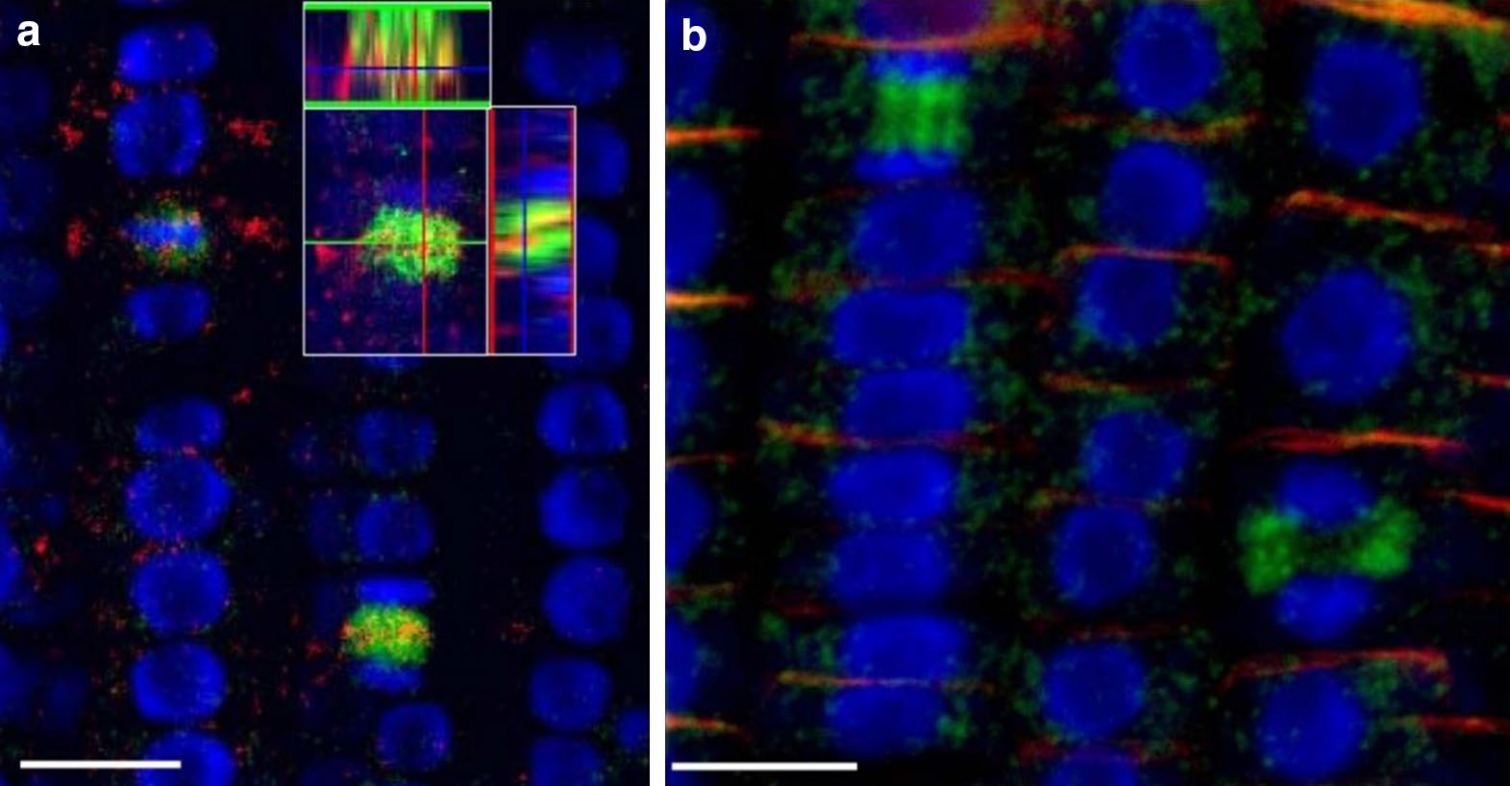
3D reconstruction of Arabidopsis root epidermis cells undergoing telophase: co-localization of β-Tubulin (TUB) and PIN1 in division plates. Four days old Arabidopsis seedlings were fixed for 30 min in formaldehyde. **a** Anti-PIN1 mouse monoclonal primary antibody (10A7) diluted 1:50 plus ALEXA Fluor ^®^ 555 anti-mouse as secondary antibody diluted 1:800 (shown in *green color*) and anti-TUB (AS10 681) rabbit primary antibody diluted 1:600 plus Goat anti-rabbit IgG (H&L), DyLight^®^ 488 Conjugate (AS09 633) diluted in 1:3000 as secondary antibody (shown in *red color*) were used. **b** Anti-PIN2 Guinea pig polyclonal primary antibody (clone A193) dilution 1:800 plus ALEXA Fluor ^®^ 555 anti-Guinea pig as the secondary antibody dilution 1:800 (show in *green color*) and anti-TUB (Agrisera, AS10 681) rabbit primary antibody diluted 1:600 plus Goat anti-rabbit IgG (H&L), DyLight^®^ 488 Conjugate (AS09 633) as secondary antibody diluted in 1:3000 (shown in *red color*) were used. Co-staining with DAPI visualizes nucleus (*blue*). *Scale bar* 20 µm. The Insertion in **a** shows an ortho-view of dividing cells

**Fig. 8:**
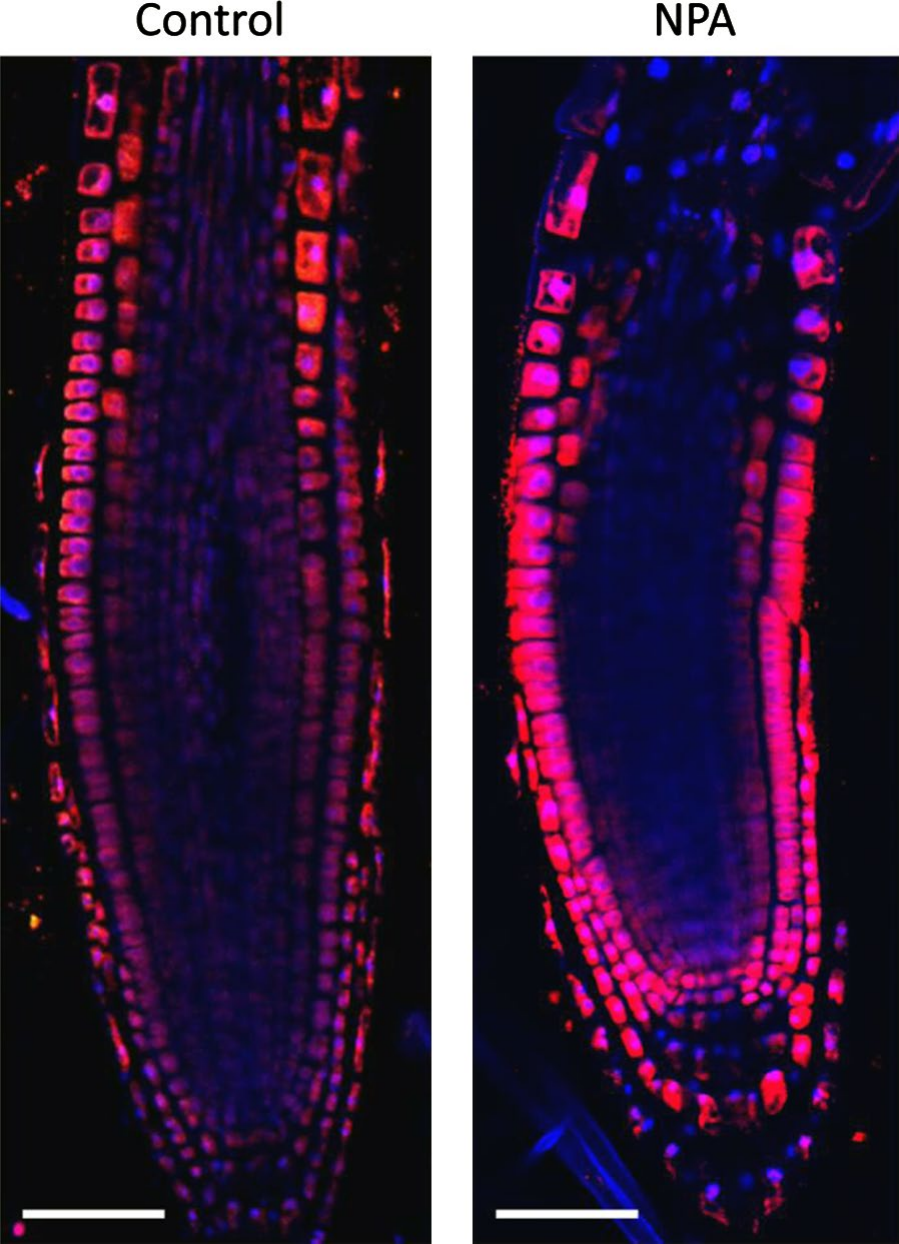
Auxin immunolocalisation in Arabidopsis roots. Four days old Arabidopsis seedlings were treated with 1 µM 1-*N*-Naphthylphthalamic acid (NPA) for 24 h to enhance accumulation of auxin in roots. Seedlings were fixed for 20 min in 4 % EDAC in 1× MTSB, and next 30 min in 4 % EDAC+ 2 % Formaldehyde. Anti-indole 3 acetic acid (IAA) rabbit primary antibody (Agrisera, AS06 193) diluted 1:600 plus Goat anti-rabbit IgG (H&L), DyLight^®^ 549 Conjugate (AS09 633) as secondary antibody diluted in 1:3000 (shown in *red color*) were used. *Scale bar* 20 µm

Previously published immunolocalization protocols [4] require at least two working days and cannot be applied to non-transparent tissues. These protocols have been applied for analysis of the root meristem of *Arabidopsis thaliana*, while for other plant species and for more dense tissues of Arabidopsis (e.g. hypocotyls or leaves) researchers prefer to use paraplast sections which are labor and time consuming and do not allow 3D reconstruction. For example, Bustos-Sanmamed et al. [2] suggested to use paraplast sections for immunolocalization in Medicago plants, which are extremely time and labor consuming. In our hands Medicago can be subjected to whole-mount immunolocalization in any organ with further 3D reconstruction. Our whole-mount protocol is applicable to the analysis of any plant species and organ including non-transparent tissues. It is also easily applicable to suspension cultures and can be completed for most specimens in 5–6 h. Detection of proteins deep inside tissues requires a fine balance between fixation, clearing of tissues, cell wall digestion and permeabilisation. Through improved tissue clearing combined with tissue-specific combinations of cell wall degrading enzymes, proteins can be detected e.g. in ovules of intact pistils or xylem-parenchyma cells of hypocotyls while keeping the outer cell structures intact (Figs. 2, 3). The excellent tissue preservation is demonstrated by labeling of microtubules and actin in the elongation zone of Arabidopsis roots (Fig. 4), which often appeared destroyed using previously published automated whole-mount method [3]. Due to the use of small volumes in Microarray slides, the procedure described here reduces the amount of reagents and limits the use of particularly precious antibodies, but also allows handling of specimens up to 1 cm wide. The general applicability of the protocol was successfully tested for localization of PIN proteins in root and flower tissues from *Medicago sativa*, *Triticum aestivum*, *Lycopersium esculentum*, and *Hedera helix* (Figs. 5, 6, 7). The fixation procedure using ethyldimethylaminopropyl carbodiimide (EDAC, carboxyl activating agent for hormones bonding with proteins) and formaldehyde was optimized for detection of low molecular weight molecules (e.g. auxin) with antibodies (Fig. 8).

**Fig. 9:**
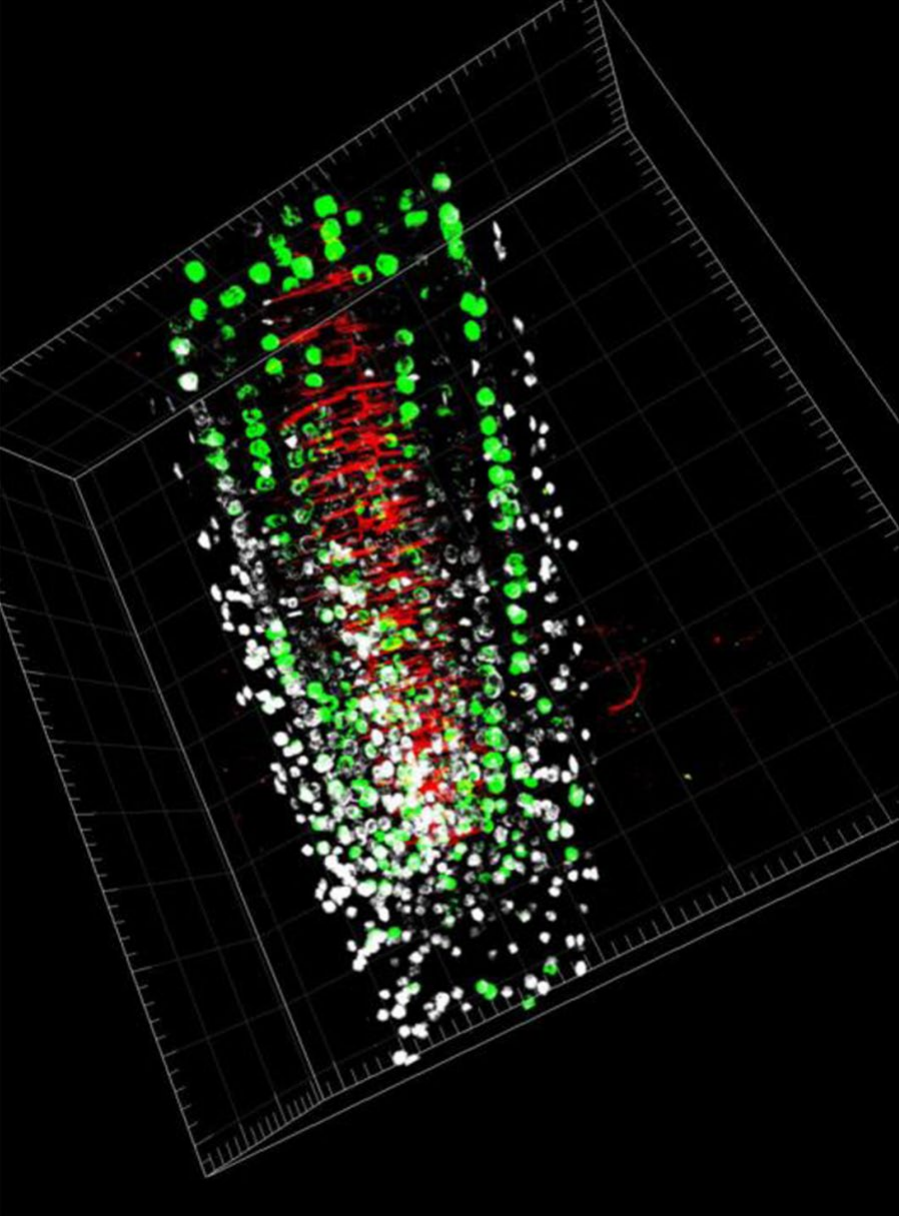
Simultaneous DNA and protein localization in Arabidospis roots. Four days old Arabidopsis thaliana seedlings were pre-cultured for 30 min in the presence of 15 μM BrdU in the *dark*. Plants were fixed in formaldehyde. Anti-PIN1 Guinea pig polyclonal primary antibody diluted 1:800 plus ALEXA Fluor ^®^ 555 anti-Guinea pig as the secondary antibody diluted 1:800 (*red color*) and mouse BrdU primary antibody (Amersham, RPN202; http://www5.gelifesciences.com) diluted 1:150 (containing DNAse) plus ALEXA Fluor ^®^ 488 goat anti-mouse IgG as secondary antibodies diluted 1:800 were used. Co-staining with DAPI visualizes nuclei (shown as artificial *color in white*). The projection of a 3D reconstruction of confocal images with IMARIS Software (Bitplane) is shown

In addition, the protocol allows further applications such as the detection of DNA replication events by using incorporation of the thymidine analogue BrdU/EdU into nuclear DNA followed by subsequent detection with an antibody recognizing BrdU/EdU (Fig. 9) [12]. This approach opens the possibility to monitor the duration of the S and G2 phases of the cell cycle, as well as to detect cells within tissues that undergo DNA reduplication. The protocol, being reasonably streamlined and simple, can be used for analysis of protein expression and localization in up to 30 samples simultaneously without the requirement of laboratory robots. As a concluding remark, our improved protocol, by keeping better intact organs structure, enables precise analysis of protein expression/localization in whole organs, thus performing a fundamental shift from two dimensional to three dimensional tissue atlases, required for our previously described automated organ analysis [9]. Examples of 3D reconstruction after immunolabelling with our protocol are shown on Additional files 3–6.

## Additional files

10.1186/s13007-015-0094-2 Agilent microarray slides suitable for immunolocalization.
10.1186/s13007-015-0094-2 Supplementary protocols.
10.1186/s13007-015-0094-2 3D reconstruction of the Arabidopsis leaf after labelling with PIN1 antibody and co-staining with DAPI for cell visualization. Four days old Arabidopsis seedlings were fixed for 30 min in formaldehyde. Anti-PIN1 mouse monoclonal primary antibody (clone 10A7) diluted 1: 50 plus Alexa Fluor^®^ 488 goat anti-mouse IgG as secondary antibody diluted 1: 800 (shown in green color) (panel A-E) were used; Co-staining with DAPI visualizes nuclei (shown as artificial color in white). Ortho-view is shown. Scale bar 50 µm.
10.1186/s13007-015-0094-2 3D reconstruction of Nicotaina tabacum roots after labelling with PIN1 antibody. Five days old Tobacco seedlings were fixed for 30 min in 2 % formaldehyde. Anti-PIN1 mouse monoclonal primary antibody (clone 10A7) diluted 1: 50 plus Alexa Fluor^®^ 488 goat anti-mouse IgG as secondary antibody diluted 1: 800 (shown in green color) (panel A) were used. Co-staining with DAPI visualizes nuclei (shown as artificial color in white) (panel B). Ortho-view was shown. Scale bar 100 µm.
10.1186/s13007-015-0094-2 3D reconstruction of Arabidopsis leaf after labelling with calcofluor white (cell wall) and propiduim iodine (nucleus). Five days old seedlings have been fixed and stained with propidium iodine (nucleus is shown in red) and calcofluor white (cell wall, displayed in green). Ortho-view was shown. Scale bar 50 µm.
10.1186/s13007-015-0094-2 Example of the automatic analysis of 3D images after EdU labelling. Five days old Arabidopsis seedlings have been incubated with EdU/colchicine for 90 min., fixed and cleared with hot methanol. Cell wall has been digested and membrane has been permeabilized. Seedlings have been incubated with EdU specific dye (C1037, Invitrogen) for 40 min., stained with DAPI and mounted on microscopic slides. Whole stacks have been scanned and 3D reconstruction has been performed using the iRoCS toolbox (http://lmb.informatik.uni-freiburg.de/lmbsoft/iRoCS). Scale bar 50 µm. Nuclei are in red; EdU are in green. Axis is in yellow.
