# Supplementary figures and images for: Protocol: an improved and universal procedure for whole-mount immunolocalization in plants
Source: Plant Methods. 2015 Oct 28;11:50. doi: 10.1186/s13007-015-0094-2 (PMC4625903; doi:10.1186/s13007-015-0094-2)

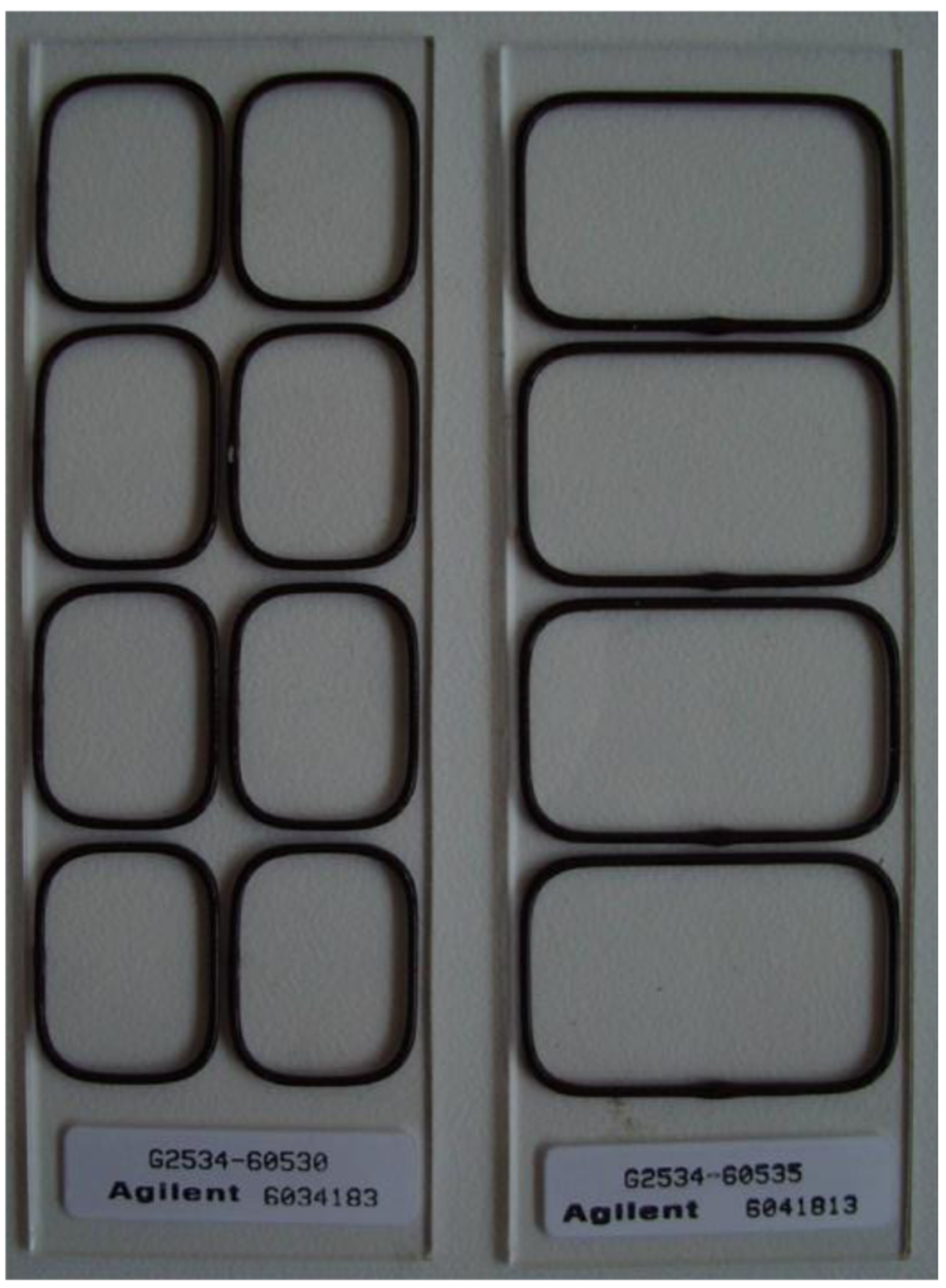

Supplement: Supplementary file 1 — 10.1186/s13007-015-0094-2 Agilent microarray slides suitable for immunolocalization. [file 13007_2015_94_MOESM1_ESM.png]

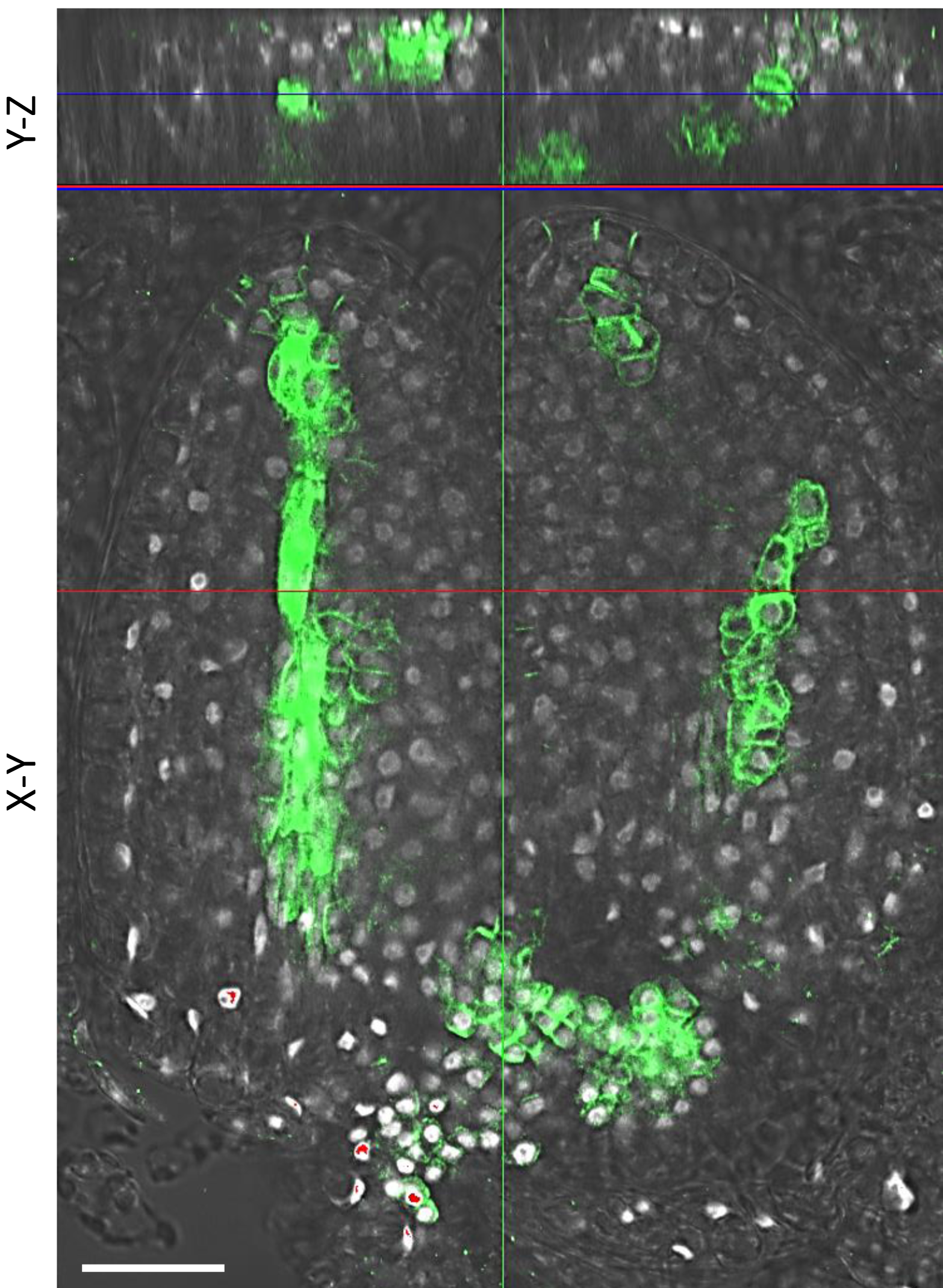

Supplement: Supplementary file 3 — 10.1186/s13007-015-0094-2 3D reconstruction of the Arabidopsis leaf after labelling with PIN1 antibody and co-staining with DAPI for cell visualization. Four days old Arabidopsis seedlings were fixed for 30 min in formaldehyde. Anti-PIN1 mouse monoclonal primary antibody (clone 10A7) diluted 1: 50 plus Alexa Fluor® 488 goat anti-mouse IgG as secondary antibody diluted 1: 800 (shown in green color) (panel A-E) were used; Co-staining with DAPI visualizes nuclei (shown as artificial color in white). Ortho-view is shown. Scale bar 50 µm. [file 13007_2015_94_MOESM3_ESM.png]

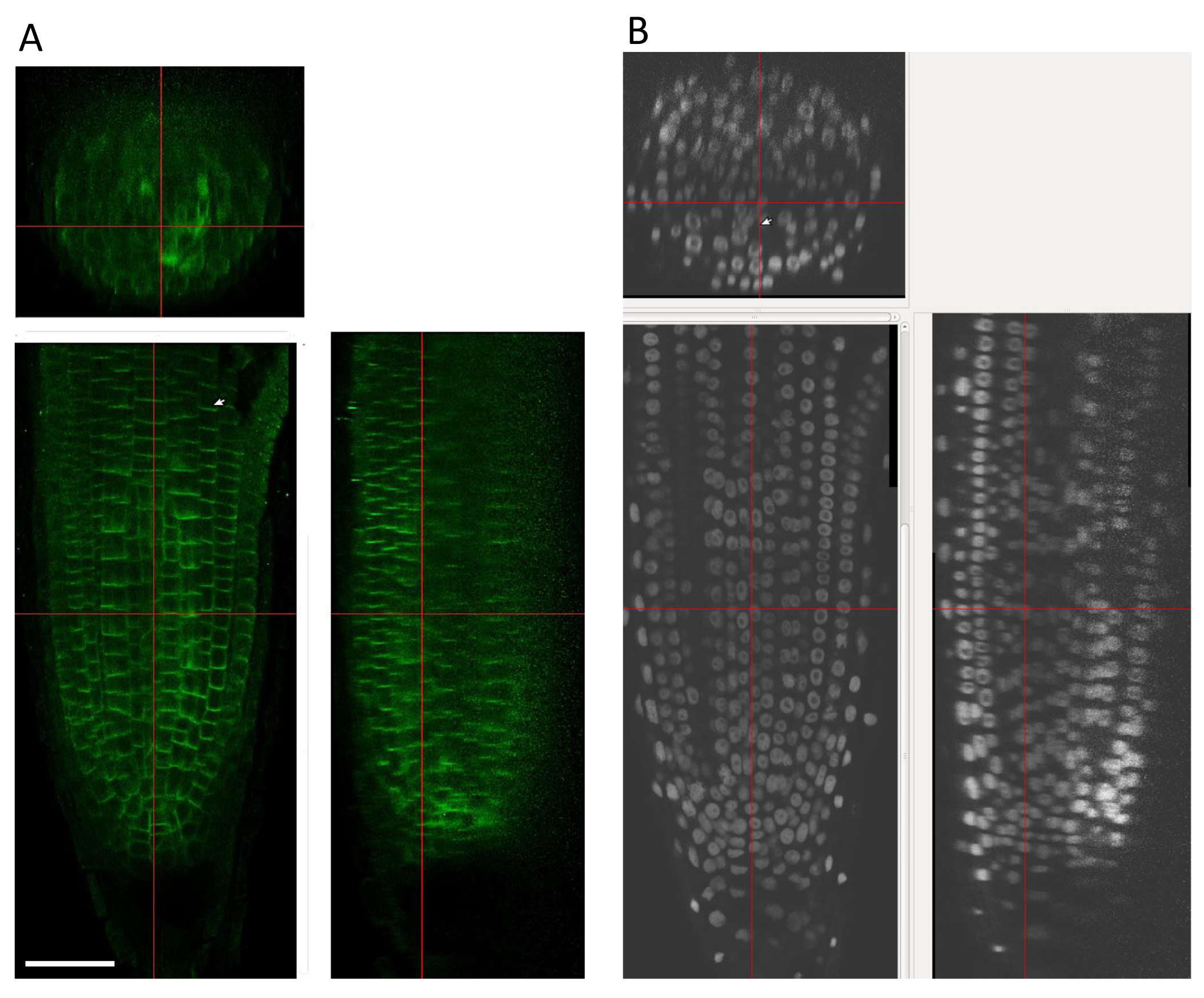

Supplement: Supplementary file 4 — 10.1186/s13007-015-0094-2 3D reconstruction of Nicotaina tabacum roots after labelling with PIN1 antibody. Five days old Tobacco seedlings were fixed for 30 min in 2 % formaldehyde. Anti-PIN1 mouse monoclonal primary antibody (clone 10A7) diluted 1: 50 plus Alexa Fluor® 488 goat anti-mouse IgG as secondary antibody diluted 1: 800 (shown in green color) (panel A) were used. Co-staining with DAPI visualizes nuclei (shown as artificial color in white) (panel B). Ortho-view was shown. Scale bar 100 µm. [file 13007_2015_94_MOESM4_ESM.png]

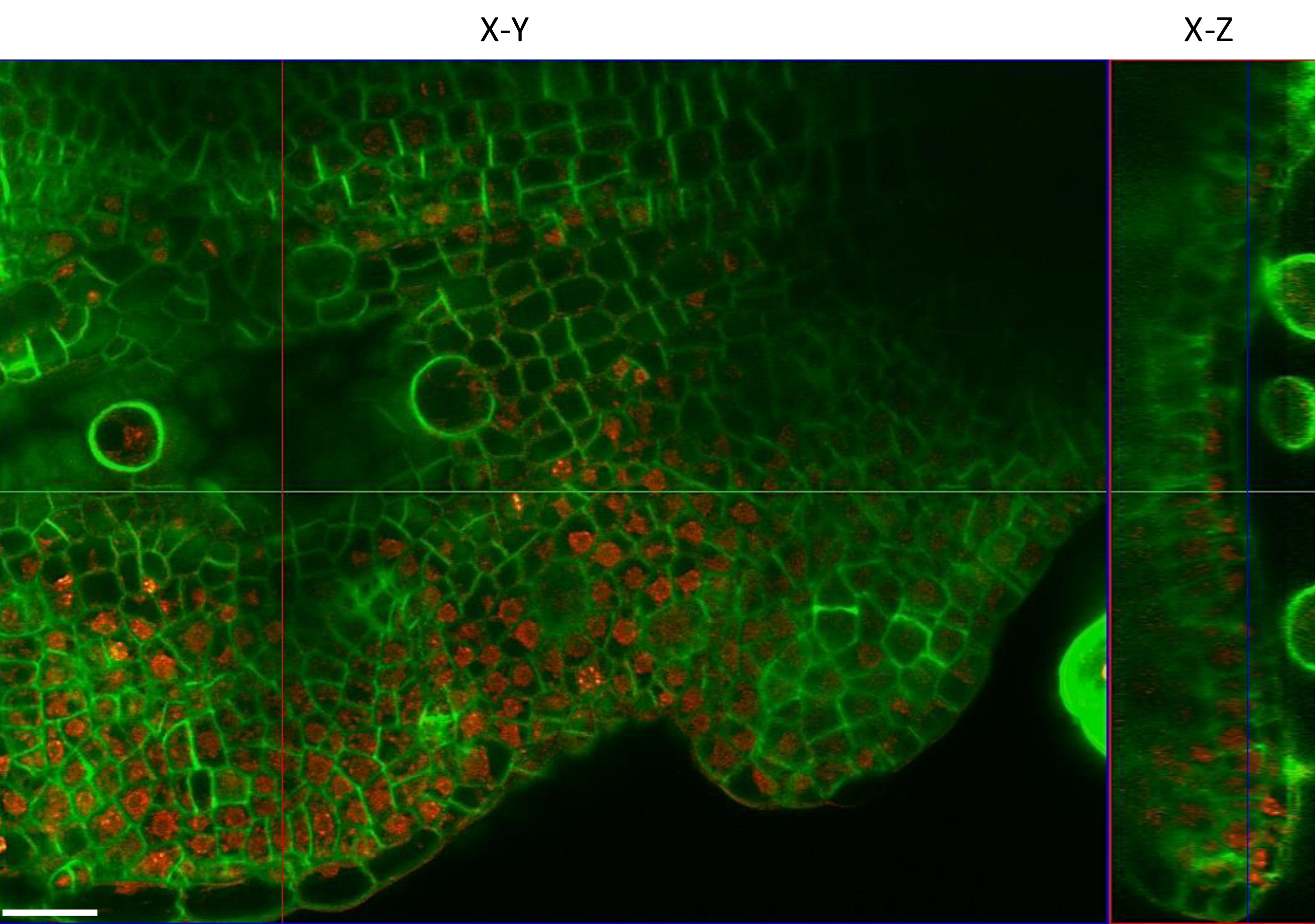

Supplement: Supplementary file 5 — 10.1186/s13007-015-0094-2 3D reconstruction of Arabidopsis leaf after labelling with calcofluor white (cell wall) and propiduim iodine (nucleus). Five days old seedlings have been fixed and stained with propidium iodine (nucleus is shown in red) and calcofluor white (cell wall, displayed in green). Ortho-view was shown. Scale bar 50 µm. [file 13007_2015_94_MOESM5_ESM.png]

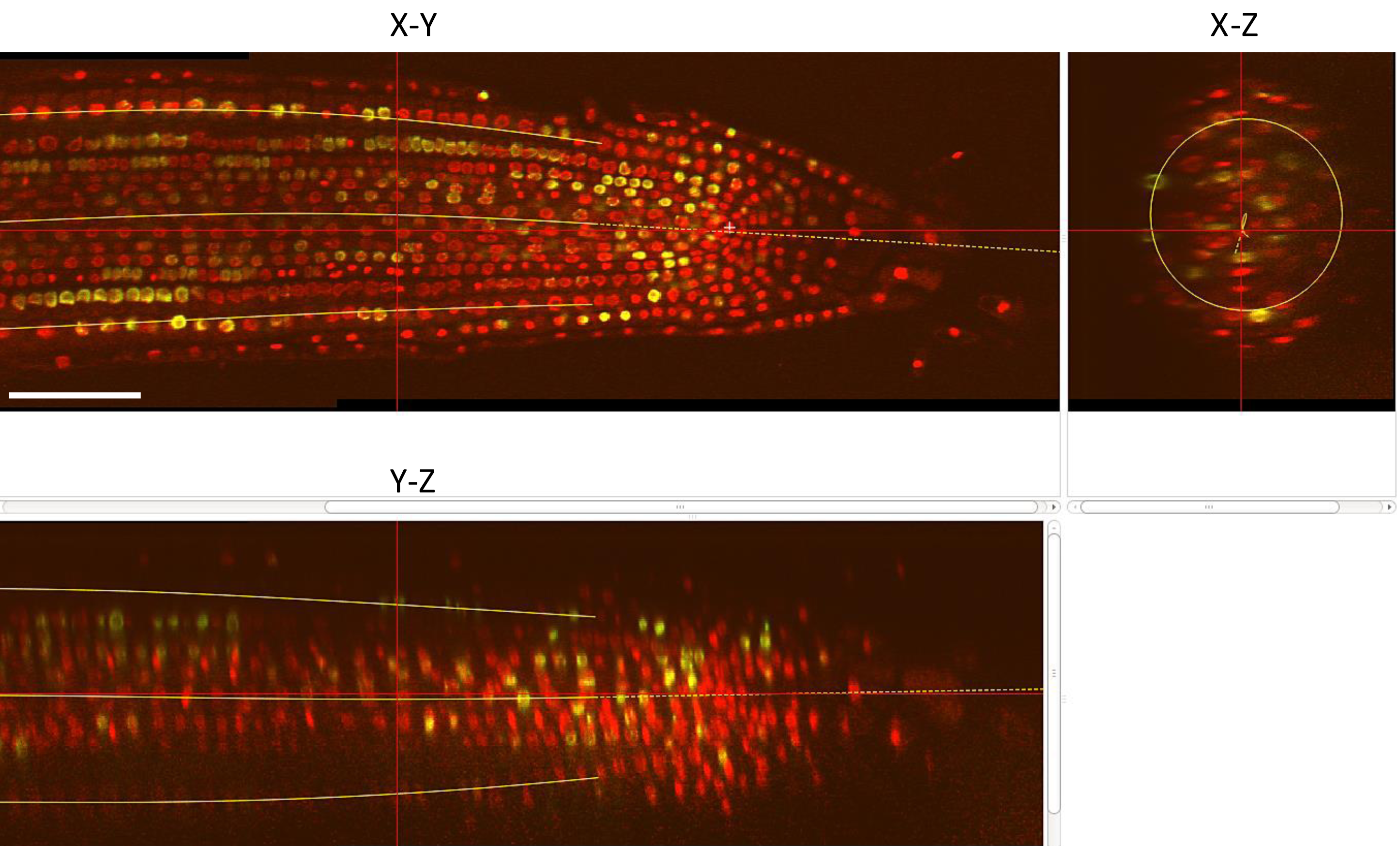

Supplement: Supplementary file 6 — 10.1186/s13007-015-0094-2 Example of the automatic analysis of 3D images after EdU labelling. Five days old Arabidopsis seedlings have been incubated with EdU/colchicine for 90 min., fixed and cleared with hot methanol. Cell wall has been digested and membrane has been permeabilized. Seedlings have been incubated with EdU specific dye (C1037, Invitrogen) for 40 min., stained with DAPI and mounted on microscopic slides. Whole stacks have been scanned and 3D reconstruction has been performed using the iRoCS toolbox (http://lmb.informatik.uni-freiburg.de/lmbsoft/iRoCS). Scale bar 50 µm. Nuclei are in red; EdU are in green. Axis is in yellow. [file 13007_2015_94_MOESM6_ESM.png]
