# Supplementary material for: Protocol: an improved and universal procedure for whole-mount immunolocalization in plants
Source: Plant Methods. 2015 Oct 28;11:50. doi: 10.1186/s13007-015-0094-2 (PMC4625903; doi:10.1186/s13007-015-0094-2)
Supplement: Supplementary file 2 — 10.1186/s13007-015-0094-2 Supplementary protocols. [file 13007_2015_94_MOESM2_ESM.docx]

### Additional File 2

### Supplementary protocols

### Supplementary protocol 1. Immunolocalization on isolated plant cells

### We provide here a modified version of the protocol optimized for the application on suspension cells, germinated pollen grain, isolated protoplasts, callus cells and protoplasts-derived colonies.

**1 - Fixation**

- Transfer samples containing cells for immunolocalization in a centrifuge tube;

- Collect cells by centrifugation in a bucket rotor at 1000 rpm for 5 min.;

- Remove culture medium and replace it with fixation buffer (1.5 ml of 2% formaldehyde in 1x MTSB);

**Critical step**: the cells/fixative ratio should be at least 1:10.

- Continue fixation for 20-25 min. under gentle shaking;

- Wash in 2 ml of 1x MTSB buffer ~ 2 times for 3 min.. Collect cells by centrifugation each time;

**Note:** Iso-osmotic buffer (1xMTSB + 0.4 M Mannitol) should be used for freshly isolated protoplasts.

**2 - Cell wall digestion**

- Add 1 ml of 0.2% Driselase and 0.15% Macerozyme containing (0.4 M Mannitol, 5 mM MES, pH 5.2 for protoplasts) to the centrifuge tubes;

**Critical step**: Since cell wall degrading enzymes require slightly acidic pH for optimal function, enzymes should be freshly prepared in water buffered with 2 mM MES, pH 5.0. Using pH 5.0 also has an additional advantage since it minimizes non-specific activities of various enzymes like DNAses or proteases, which are present in the Driselase/Macerozyme and have optimum at pH 7.

**Note:** enzyme solution can be prepared in small aliquots (2-3 ml) and kept frozen at -20°C until use. Enzyme concentrations might have to be optimized for each tissue and cell type. For the immunolocalization of proteins in protoplasts iso-osmotic buffer should be used.

- Incubate for 10-15 min. at 37°C;

- Wash 2 x 5 min in 1x MTSB pH 7.0;

**3 - Membrane permeabilization**

- Load cells on poly-L-lysine coated slides and let them dry completely;

**Note:** this procedure replaces chemical membrane permeabilization.
- After completely drying cells have to be rehydrated with 1x MTSB buffer for 10 min.

**- Alternatively:**

- cells can be loaded on poly-L-lysine coated slides, kept for 5-10 min. till they settle and stick to the slides. Then buffer should be carefully removed and membrane permeabilization solution (1-2% IGEPAL C630, 3-5% DMSO in 1x MTSB) should be added;

- incubate for 10 min. at 37°C;

- wash 4 times in 1x MTSB for 5 min. each;

**4 - Incubation with primary antibody**

- Add blocking buffer to the slides and incubate for 10-15 min at 37°C;
- Replace BSA blocking solution with primary antibody solution and incubate for 1-2 h at room temperature;
- Wash 2 x 3 min in 1x MTSB;

**Critical step**: Do not mix the solution during incubation with the primary antibody.

**5 - Incubation with secondary antibody**

- Add secondary antibody solution and incubate for 1 hour at 37°C;

- Wash 1 x 5 min in 1x MTSB;

**Critical step**: Do not mix (stir) solution during incubation with the secondary antibody.

**6 - DAPI staining**

- Add DAPI containing solution (0.03 mg/l) and incubate for 5-10 min.;

- wash 2 x 2 min. with 1x MTSB;

**7 - Mounting**

- Remove the buffer;

-Add 20-40 μl of Fluoromount G or other mounting medium and carefully cover cells with cover slips;

**Comments:** DAPI should be dissolved in water at a concentration of 1 mg/ml and diluted just before usage as 0.3 μl/10 ml; 1 mg/ml stock solution is stable for a long time and should be kept at 4°C in the dark.

**Supplementary protocol 2. Labelling and 3D-imaging of whole plant organs.**

Here we provide a short version of the protocol for investigation of 3D structure of plant organs, including very dense leaves of several plant species.

Proceed to fixation, hot methanol treatment, cell wall digestion and permeabilization as for immunolocalization rpotocol.

Thereafter, the following procedure must be followed:

- Incubate in 10 mM Tris-HCl, pH 7.5 for 10 min.;

- Incubate in 100 µl of the RNAse A solution (10 mg/l) in 10 mM Tris-HCl, pH 7.5 for 30 min. at 37°C;

- Wash 1x5 min with 100 µl of 10 mM Tris-HCl, pH 7.5;

- Incubate in 100 µl of propidium iodine solution (0.4 mg/l) in 10 mM Tris-HCl, pH 7.5 for 10 min. at 37°C;

- Wash with water for 10 min.;

- Incubate in 100 µl of 10 mM Tris-HCl, pH 9.2 for 10 min.;

- Incubate in 100 µl of calcofluor white solution (10 mg/l from 10 mg/ml stock in DMSO) in 10 mM Tris, pH 9.2 for 20 min.;

- Wash 2x5 min. in the 10 mM Tris-HCl, pH 9.2.

- Mount in alkaline antifade medium by mixing of 70% of antifade medium with 30% of 500 mM Tris-HCl, pH 9.2 (350 µl antifade medium + 150 µl 500 mM Tris-HCl, pH9.2).

An example of the labelling procedure is shown on Additional file 5.

**Supplementary protocol 3. Usage of 3D images for analysis of gene expression.**

Scan images as 3D stacks with at least one protein of interest labeled or EdU labeled and one marker channel (nucleus or cell border).

- Convert images into .hdf5 and load into iRoCS software (http://lmb.informatik.uni-freiburg.de/lmbsoft/iRoCS)[9].
- Perform coordinates attachment (segmentation/nucleus detection).
- Perform detection of the patterns of the protein of interest/Edu localization.
- Extract necessary data according to [9].

Example of this type of analysis is shown on Additional file 6.
